# Air Quality Sensors Systems as Tools to Support Guidance in Athletics Stadia for Elite and Recreational Athletes

**DOI:** 10.3390/ijerph19063561

**Published:** 2022-03-17

**Authors:** Mar Viana, Kostas Karatzas, Athanasios Arvanitis, Cristina Reche, Miguel Escribano, Edurne Ibarrola-Ulzurrun, Paolo Emilio Adami, Fréderic Garrandes, Stéphane Bermon

**Affiliations:** 1 Institute of Environmental Assessment and Water Research (IDAEA-CSIC), 08034 Barcelona, Spain; cristina.reche@idaea.csic.es; 2 Environmental Informatics Research Group, School of Mechanical Engineering, Aristotle University, 54124 Thessaloniki, Greece; kkara@auth.gr (K.K.); thanos.k.arvanitis@gmail.com (A.A.); 3 Kunak Technologies, 31160 Orcoyen, Spain; mescribano@kunak.es (M.E.); eibarrola@kunak.es (E.I.-U.); 4 Health and Science Department, World Athletics, 98000 Monaco, Monaco; paoloemilio.adami@worldathletics.org (P.E.A.); frederic.garrandes@worldathletics.org (F.G.); stephane.bermon@worldathletics.org (S.B.); 5 Laboratoire Motricité Humaine Expertise Sport Santé (LAMHESS), Université Côte d’Azur, 06000 Nice, France

**Keywords:** air pollution, competitions, exposure, stadiums, track and field, training, performance, exercise

## Abstract

While athletes have high exposures to air pollutants due to their increased breathing rates, sport governing bodies have little guidance to support events scheduling or protect stadium users. A key limitation for this is the lack of hyper-local, high time-resolved air quality data representative of exposures in stadia. This work aimed to evaluate whether air quality sensors can describe ambient air quality in Athletics stadia. Sensing nodes were deployed in 6 stadia in major cities around the globe, monitoring NO_2_, O_3_, NO, PM_10_, PM_2.5_, PM_1_, CO, ambient temperature, and relative humidity. Results demonstrated that the interpretation of hourly pollutant patterns, in combination with self-organising maps (SOMs), enabled the interpretation of probable emission sources (e.g., vehicular traffic) and of atmospheric processes (e.g., local vs. regional O formation). The ratios between PM size fractions provided insights into potential emission sources (e.g., local dust re-suspension) which may help design mitigation strategies. The high resolution of the data facilitated identifying optimal periods of the day and year for scheduling athletic trainings and/or competitions. Provided that the necessary data quality checks are applied, sensors can support stadium operators in providing athlete communities with recommendations to minimise exposure and provide guidance for event scheduling.

## 1. Introduction

The different links between air pollution and cardiovascular and respiratory disease are well established [1,2,3,4,5], especially for general and high-risk populations. Research is also available on health impacts for general populations performing physical activities in urban environments (walking, cycling; [6,7,8,9,10,11]). However, the literature is scarce on recreational and elite athletes’ exposure to air pollution [12,13]. Indeed, this population is at higher risk due to the increased ventilation during exercise, which in is addition highly variable as a function of the type of exercise (e.g., sprint, endurance, team sports, etc.; [14]). This is explained by the greater fraction of air inhaled during exercise, effectively bypassing the normal nasal filtration mechanisms in the case of particles, and the increased airflow velocity, carrying pollutants deeper into the respiratory tract [15,16,17,18].

In addition to the impacts on health, exposure to air pollution may also decrease athletes’ performance [16,19], a consequence which is receiving increasing attention from sport governing bodies due to subsequent implications in terms of public image and loss of revenues [20,21,22]. Poor air quality deriving from conventional (e.g., traffic) and climate-driven emerging sources (e.g., wildfires) has impacted major sports events in recent years (Athens 2004 and Beijing 2008 Olympics; 2020 Australia Tennis Open, etc.; [16,21,23]), something which will only increase in the future [24]. National and regional events were also postponed, such as an Australia Football Federation W-league match (January 2020), or a football game between the University of California, Berkeley and Stanford University during the 2018 California wildfire season. Some professional sport leagues (e.g., US National Women’s Soccer League) which maintained competitions despite smoky air, and had to implement mitigation measures such as extra hydration breaks and oxygen on-hand at the sidelines, faced criticisms for insufficiently addressing the issue [23]. Moreover, taking into account the non-negligible percentage of asthmatic and allergic subjects in the athlete community [17], the influence of air pollution on athletes’ health and performance becomes even more important as it increases the effect of aeroallergens and also has negative synergistic effects with asthma triggering parameters. Aside from sport governing bodies, the demand for air pollution information is also rising from recreational athletes in urban areas (e.g., cyclists and runners), as suggested by the increasing number of apps linking GPS tracking with air pollution data. Several private sector initiatives have also started to focus on air quality monitoring around sports facilities (e.g., https://around.uoregon.edu/content/hayward-field-sensors-advance-uo-wildfire-smoke-initiative (accessed on 6 February 2022); https://learn.kaiterra.com/en/resources/case-study-kaiterra-atmosair-help-us-bank-stadium (accessed on 6 February 2022)).

Climate change-driven air pollution is now putting the sports world under increasing pressure. Despite this emerging health concern, guidelines to minimise air pollution exposures in sports are rare and official recommendations (from event organizers as well as local authorities) are mostly non-existent [25,26] (https://www.ncaa.org/sports/2017/9/14/air-quality.aspx (accessed on 6 February 2022)). Environmental parameters policies, such as heat or cold policies, are available from several national and international sports governing bodies and major tournament organisers but guidance does not exist for air quality. Some sports organizations have started looking into the issue, mostly providing generic advice (e.g., https://footballnsw.com.au/2019/11/21/the-impact-of-poor-air-quality-and-high-temperatures-on-the-football-player/ (accessed on 6 February 2022)). For instance, World Athletics (previously known as International Association of Athletics Federations, IAAF), monitors air quality on a routine basis during World Championships [13] as a part of its sustainability strategy since 2019 [27].

The lack of high-quality air pollution data in the competition venues or their immediate vicinity is currently a limiting factor for exposure characterisation. This limitation also exists for other locations of interest (e.g., hospitals, schools, elderly homes; [28]), and is linked to the fact that siting of reference air quality monitoring stations(EU Air Quality Directive) aims to represent average population exposures (as opposed to specific locations). Significant differences have been reported in the literature [29,30,31] between air pollutant concentrations monitored at central locations and personal exposures. To overcome this issue and increase the spatial resolution of air quality mapping, sensor technologies have developed, evolving from significantly poor performances in early years to the current applicability of sensors when sufficient data quality checks are implemented and following the “fit for purpose” approach [32,33,34,35,36,37,38]. Research is abundant on testing the performance of diverse sensor technologies (individual sensing components as well as sensing nodes; [33]) under different conditions in the laboratory and in the field (see [38] and references therein), by comparison with high quality, reference data from nearby locations [39,40]. However, air quality sensors are also available on the market for any type of target user (e.g., consumers; small urban agglomerations) who may use sensor technologies with no prior validation or scientific guidance as to their performance. The CEN working group WG42 is dedicated to standardised guidance for sensor technologies [41], and tools are being developed to assess compliance of sensor technologies with the data quality objectives of the EU Air Quality Directive [42]. The literature evidences that electrochemical and optical sensors suffer from limitations [32,34,35,36,43,44,45,46,47] (among others): data are affected by ambient conditions, are susceptible to cross-sensitivities and may suffer from drifts over time, and thus require calibration under local ambient and aerosol mix conditions to ensure data quality. Recent reviews report on diverse applications of air quality sensors, ranging from personal exposure assessment to source interpretation on the basis of diurnal and seasonal patterns [48,49,50]. As discussed by [51], there is no single widely-accepted definition of the price categorisation of air quality sensors, as available categorisations are based on different variables (cost of the individual device, cost of the final nodes, target users e.g., consumer-based vs. research-based, etc.) [34,52]. Taking different approaches into account, the sensing units used in this work are referred to as medium-cost air quality monitors (MAQM).

Given the growing interest of sports organisations in air quality and the lack of hyper-local data recorded inside stadia, the aim of this work was to showcase the use of MAQMs in Athletics stadia for air quality and exposure assessments. The goal was to evaluate the extent to which these monitors can contribute to air quality characterisation when deployed off the shelf, without local validation against reference air quality data, as this is frequently the way in which this kind of sensor is used outside the scientific community. While it is evident that local calibration and quality control are essential to ensure that high-quality data are produced, in practice, it is frequently the case that these monitors are used lacking local calibration, by users outside the scientific community. As a result, this work aimed to understand the performance of MAQMs under these conditions and to underpin their limitations. The expected practical applications of this work are to (i) support stadium operators in providing recreational athlete communities with recommendations to minimise exposure to air pollutants, and (ii) guide athletics competition organisers in their event scheduling. The assessment of links between air quality and athlete performance or public health are outside the scope of this work and will be addressed in future research.

## 2. Materials and Methods

### 2.1. Monitoring Locations, Instrumentation, and Strategy

A pilot study was initiated in 2018 by World Athletics which deployed 6 identical units of a commercial MAQM (KunakAir, Kunak Technologies, Pamplona, Spain) simultaneously in the main athletics stadia of 6 major cities around the globe, over a 1-year period. The cities and exact locations cannot be reported due to confidentiality issues, which is considered a limitation of the study. However, it should be noted that the aim of our study is not to compare locations with each other. One monitor was installed in Europe, one in Central America, one in Asia, one in Oceania, and two in Africa (Table 1). The locations represented a variety of climatic conditions (from coastal to inland, central to Southern latitudes). All of the stadia were located in major urban areas and sited in typically urban environments, surrounded by major and medium-sized roads. Stadia #1, #3 and #4 were in coastal cities, while cities #2, #5 and #6 are found inland and at altitudes of 2300, 2200 and 1800 m a.s.l., respectively. All monitors were deployed inside open stadia, at ventilated locations around the perimeter of the track area (between the track and the spectator seating area), and at heights varying between 1.5 m and 4 m above ground. Thus, they were considered representative of the exposure of athletes and visitors sitting along the perimeter of the tracks during competition and training. The observation periods differed in each case due to technical issues at each location but were at of 5 months (stadium #6; Table 1). The resulting dataset accounted for almost 200.000 valid data points with a 5 min time resolution.

The monitors integrate Alphasense sensors for PM_1_, PM_2.5_ and PM_10_ (OPC–N3), NO_2_ (NO_2_–B43F), NO (NO-B4), CO (CO-B4), and ozone (O_3_; OX-B431), temperature and relative humidity. The following calibration process was applied:

1. The 6 monitors were co-located by the manufacturer over a 15-day period at an EU-reference station from the air quality monitoring network in Navarra (Spain), against which they were calibrated following internal standard procedure. The sensors contain a property algorithm to correct for temperature and humidity influence and gas cross-interference. According to an independent evaluation carried out by the US South Coast AQMD (Air Quality Sensor Performance Evaluation Center, AQ-SPEC, http://www.aqmd.gov/aq-spec# (accessed on 6 February 2022)), the relative intra-model variability (calculated as the absolute intra-model variability relative to the mean of the three sensor means) of Kunak monitors (3 units tested) was 1% for O_3_, 11% for NO, 3% for NO_2_, 66% for CO, 13% for PM_2.5_ and 10% for PM_10_ (http://www.aqmd.gov/docs/default-source/aq-spec/field-evaluations/kunak-air-a10---field-evaluation.pdf?sfvrsn=24 (accessed on 6 February 2022)). Comparability across the six units used in this study was subsequently assumed, based on this independent evaluation.
2. One of the monitors (#1) was deployed at the Barcelona EU-reference air quality monitoring station in Palau Reial, where its performance was compared over a 5-day period to that of EU-reference, research-grade instrumentation. The results are presented in Figure S1 in Supporting Information and Section 3.1. This unit was, at the time, only equipped with sensors for gaseous pollutants. The performance of PMx sensors was validated for another of the units (#5) when it arrived at its destination, where it was possible to co-locate it at a local reference station following non-EU national standard quality procedures, during 3 months (Figure S2 in Supporting Information, and Section 3.1). These intercomparisons were only applied to two of the monitors due to logistical reasons and under the assumption of comparability across units, as described above. It should be noted that the calibration parameters for these nodes were not modified after the comparison so that they remained comparable to the rest of the monitors.
3. Finally, the monitors were shipped to their respective stadia and installed by local staff. Once at their destinations, the units were not calibrated against local air pollutant or meteorological reference data, given that access to local reference data was not available at all locations. As discussed above, the purpose of this work was to understand the potential use of sensor data when deployed by users outside the scientific community, and potentially with little to no previous knowledge of the air quality concentrations in the study area, following the “from the shelf to the field” use. Because the monitors were not calibrated locally, the absolute concentrations of particulate and gaseous pollutants monitored should not be used for compliance checking and/or comparisons across cities [38].

Reference data from local air quality networks were available in locations #1, #3 and #5. City #4 has a local air quality network, although the data were not accessible. Finally, cities #2 and #6 did not officially monitor air quality at the time of this work [53].

### 2.2. Data Analysis Methods

The mean daily evolution of concentrations of particulate matter (PM_10_, PM_2.5_, PM_1_), gaseous pollutants (NO, NO_2_, O_3_, CO) and meteorological variables (temperature, T; relative humidity, RH) were calculated and plotted, to identify mean daily hourly trends, profiles, and similarities among parameters. The results are presented in terms of hourly and monthly mean concentrations, with a 95% confidence interval (CI). Moreover, the PM_2.5_/PM_10_ ratio was assessed using box plots for the full monitoring period in each location, aiming at identifying similarities and differences concerning the particle size distribution among the study areas, and therefore identifying possible differences in relevant emission sources.

In addition, the Self Organizing Maps (SOMs) method was used to identify relationships and dependencies among parameters monitored per location [39,54]. SOMs are an unsupervised machine-learning algorithm that receives multi-dimensional input and transforms it in a low dimensional space representation (usually 2D) visualised as a graph (i.e., a map). The method is based on artificial neural networks (ANNs) and employs a two-dimensional array of (initially) randomly weighted neurons [55,56]. The main difference with ANNs is that SOMs apply competitive learning instead of trying to minimise an error function, and they also employ a neighborhood function which allows to map vectors of the input space characterised by a certain similarity, in the same “neighborhood” of the SOM (preservation of topological properties of the input space). All data points (input vectors) are passed through the neural network and are matched with a winning neuron, causing the network topology to adjust and eventually form clusters of similar attributes, while weights are updated to better fit into the process. The unified distance matrix (U-matrix), commonly used for SOM visualisation, represents the Euclidean distance between neighbouring neurons which is actually an expression of the relationship (i.e., the “similarity”) between neighbouring neurons (Ultsch and Siemon, 1990): the highest values for a specific SOM location in the U-matrix, correspond to a “borderline” between areas of the SOMs, indicating clusters of values. This similarity is used as a criterion to reveal potential relationships and dependencies between parameters of the input space: after constructing a SOM for each parameter, areas of the map for each parameter are considered to belong to the same neighbourhood. This may be interpreted as a suggestion of a relationship between the aforementioned parameters, which may refer to relationships between pollutants (e.g., the inverse relationship between O_3_ and NO_2_) but also to potential artefacts (e.g., interference of RH on monitored PM concentrations). In addition, SOMs can contribute to the identification/confirmation of emission sources impacting pollutant concentrations in the stadia, and as an internal quality control mechanism by comparison with the results from the time-series analysis. SOMs were calculated with the aid of the SOM Toolbox made available via the Aalto University [57].

## 3. Results and Discussion

### 3.1. Comparison with Reference Data Prior to Deployment

The results from the comparisons between monitor#1 and EU-reference data (in Barcelona, Spain), and monitor #5 with national reference data (in city #5) are shown in Figures S1 and S2 in Supporting Information. The instrumentation at the Barcelona station was EU-reference analysers for gases and an EU-equivalent monitor (GRIMM laser spectrometer) corrected against EU-reference gravimetric measurements. As described above, these results were obtained based on the monitors’ initial calibrations, which were not modified after the comparison. Results evidenced statistically significant comparability between sensor and reference data for NO_2_ (R^2^ = 0.90), O_3_ (R^2^ = 0.85) and NO (R^2^ = 0.89), while the correlation was lower for PM_10_ (R^2^ = 0.70) and PM_2.5_ (R^2^ = 0.82), and even lower for CO (R^2^ = 0.41). The resolution of the CO reference instrument and the sensor were not directly comparable, and the reference CO monitor suffered from frequent data losses. The combination of these factors resulted in a poor performance of this sensor. Overall, the monitors underestimated air pollutant concentrations, by approximately 10% (for NO_2_) and up to 60% (for CO).

### 3.2. Time Series Analysis

Mean daily trends were calculated and plotted for all pollutants, for a period when data were available for all stadia. The purpose was to compare hourly trends in view of source identification, considering the meteorological and seasonal variability across stadia.

#### 3.2.1. Meteorological Variables

Despite the seasonal differences across hemispheres, the daily trends of ambient temperature and relative humidity (Figures S3 and S4 in Supporting Information) were mostly comparable across the 6 locations, with the main differences being registered in terms of maximum temperatures (ranging between 21 °C and 35 °C) and minimum temperatures (11–27 °C). The same was true for ambient relative humidity, with only an earlier and narrower dip in the midday hours for stadium#1. On average, ambient relative humidity was highest in stadia #4, #5 and #6, which may be relevant as the influence of relative humidity on sensor performance, especially in terms of PM sensors, is well documented [38,44].

#### 3.2.2. Gaseous Pollutants

**Figure 1:**
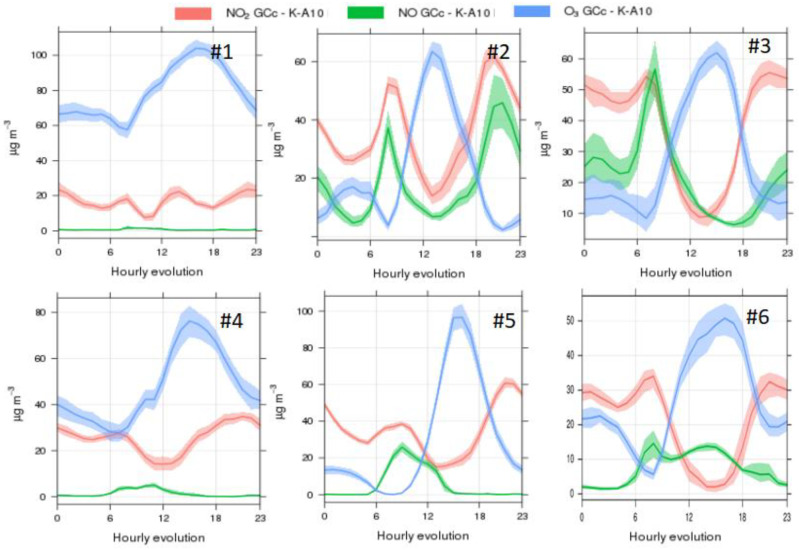
Mean daily evolution of gaseous pollutants NO_2_, NO and O_3_ for the months July–September 2019, for the 6 stadia in major cities in: #1: Europe, #2: Africa, #3: Oceania; #4: Asia; #5: America; #6: Africa (data from October–December 2020).

**Figure 2:**
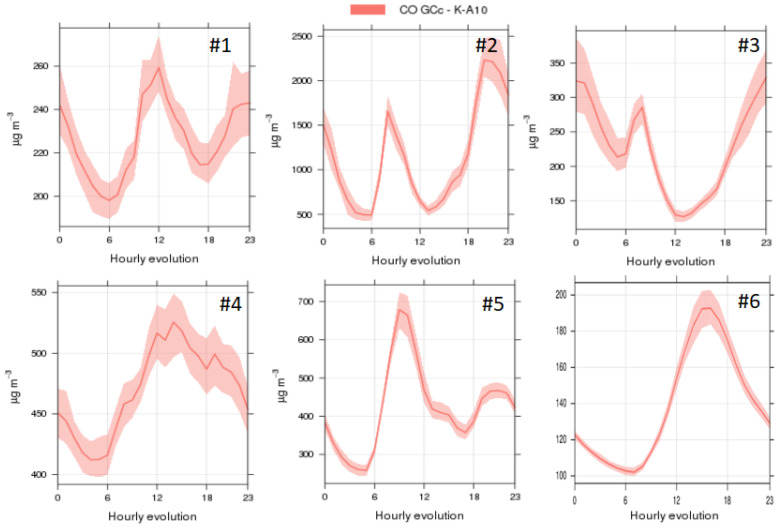
Mean daily evolution of CO for the months July–September 2019, for the 6 stadia in major cities in: #1: Europe, #2: Africa, #3: Oceania; #4: Asia; #5: America; #6: Africa (data from October–December 2020).

Air pollutant concentrations showed different patterns across stadia. Despite varying intensities, all stadia seemed to be influenced by vehicular emissions from the surrounding urban areas, based on the daily patterns recorded (Figure 1 and Figure 2). Gaseous pollutant concentrations (NO, NO_2_) followed the characteristic traffic pattern, with peaks during the morning and evening rush hours from vehicle exhaust (roughly, 6–9 h and 18–22 h, in the different stadia), which was inverse to the pattern monitored for O_3_ (Figure 1) as expected due to titration [58,59]. The pattern was stronger in stadia #2, #3 and #6, located closest to major roads in each city, but it was also detectable (in the form of a morning peak) in #1, #4 and #5, indicating that stadium users are exposed to vehicular traffic emissions during Athletics practice. While two of the stadia (#2 and #3) showed two daily NO peaks (morning and evening), in others (#1, #4, #5) only the morning peak was detected. Stadium #6 was characterised by an unusual midday NO relative peak. CO concentrations (Figure 2) did not follow such repetitive trends as in the case of NO and NO_2_, possibly pointing to the poorer performance of the CO sensors (Figure S1).

#### 3.2.3. Particulate Pollutants

**Figure 3:**
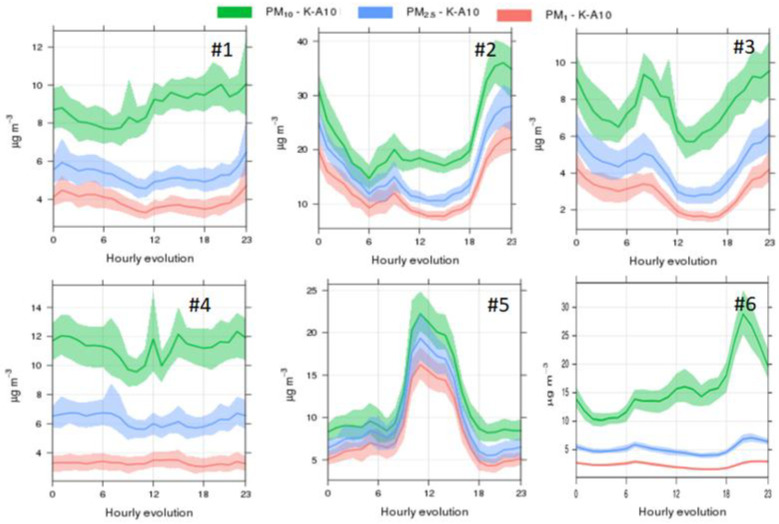
Mean daily evolution of PM_10_, PM_2.5_ and PM_1_ for the months July–September 2019, for the 6 stadia in major cities in: #1: Europe, #2: Africa, #3: Oceania; #4: Asia; #5: America; #6: Africa (data from October–December 2020).

Particulate matter concentrations, on the other hand (Figure 3), showed markedly different trends across stadia. Once again, the characteristic traffic pattern was evident in two of the locations (#2 and #3), with varying intensities of the morning (in stadium #3) and evening (#2) peaks. It should be noted, however, that source identification was carried out based solely on temporal trends and that additional information (e.g., tracers such as black carbon, ultrafine particles, particle chemical characterisation) was not available from the monitoring locations. The patterns observed in stadia #1 and #4 did not suggest the influence of specific sources, with slight increases in PM concentrations in the afternoon hours (>15 h). Stadium #6 reported a major increase towards the end of the day (>18 h), especially impacting coarse particles, which coincided with the second NO_2_ increase in the day and may thus could be related to evening traffic. The low PM_2.5_ and PM_1_ concentrations recorded, decoupled from those of PM_10_, could evidence the impact of a specific coarse particle source in this stadium. Finally, the trend observed in stadium #5 was highly unusual and it would point to the influence of a PM source impacting mostly during the midday hours. This trend was compared with data from a reference station from the same city, located approximately 20 km from the stadium (Figure S5), with the aim to understand the representativity of this daily pattern. Results suggested that the patterns are indeed representative of urban air despite the significant distance between both locations and that this relatively unusual daily pattern was driven by emission sources or meteorological characteristics which seem to be specific to this location and should be further investigated.

The mean daily variability of air pollutant concentrations described above could be used by event organisers to minimise air pollutant exposures during competitions. For example, in the case of athletes with an asthma condition, typically triggered by exposure to high O_3_ concentrations [60,61,62], it would be advisable for them to avoid training between 14–19 h in stadium #1, while the most polluted time window would be earlier in the day (between 11–16 h) if they were training in stadium #2 (for the period July–September, Figure 1). Similarly, athletes or recreational sports practitioners with a heart condition (impacted by exposure to fine particles; [63,64,65]) should be advised to avoid training between 10–17 h in stadium #5, whereas this would be the optimal time of the day (i.e., with the lowest PM concentrations) for training in stadium #2 (between July and September; Figure 3).

In addition, the concept of having hyper-local data at the stadia (either from reference instrumentation or locally calibrated sensors) would allow setting guidelines regarding potential thresholds above which events should be postponed or cancelled. Potential thresholds could be set in terms of absolute pollutant concentrations which should not be exceeded on competition days (if reference instrumentation is used), or by defining ratios for air pollutants between competition days and the days prior to the competition (e.g., a comparison for PM_2.5_ between the day of the competition and the previous week should not exceed a certain threshold). The increasing impacts of poor air quality on international sports events point to the need for internationally agreed guidelines to safeguard athletes’ respiratory health and avoid inequalities in training and/or competing conditions.

**Figure 4:**
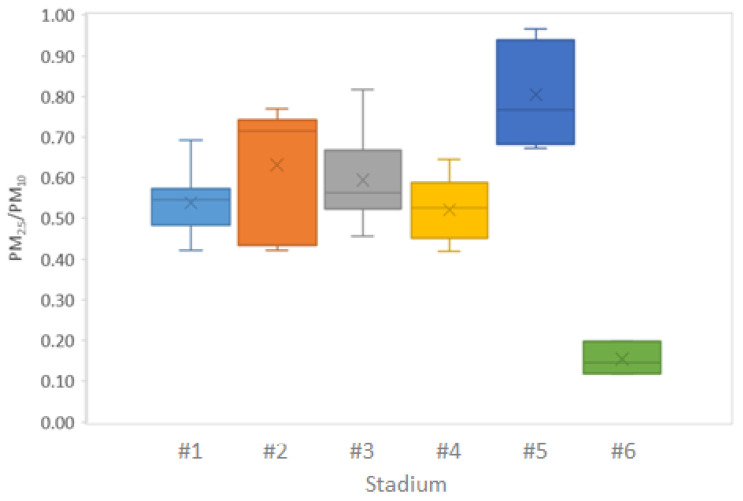
Mean PM_2.5_/PM_10_ ratios for the full monitoring period in each location.

In addition to hourly trends, the particle size distribution was assessed in each city in terms of the PM_2.5_/PM_10_ ratio (Figure 4). The differences observed may indicate differences in emission sources. For the respective full monitoring periods, average PM_2.5_/PM_10_ ratios were relatively similar in stadia #1, #3 and #4 (0.52–0.59), also with relatively limited intra-annual variability (standard deviation = 0.07–0.11). A similar ratio was expected for stadium #2, where a higher average was obtained due to the lack of data (technical issues with the monitor) during most of the dry season in this African location (February through April). This resulted in artificially high PM_2.5_/PM_10_ ratios, representative mostly of the wet season (0.60–0.77) with minimal dust resuspension, as opposed to the dry season (PM_2.5_/PM_10_ = 0.42–0.44). The daily patterns of gaseous pollutants (NO, NO_2_, O_3_), together with the similarity in particle size distribution, suggest that traffic could be the main air pollution source impacting the stadia at these locations. However, other combustion sources such as residential combustion cannot be discarded. Conversely, in stadium #5 (in the American continent), the average ratio obtained (0.80, ranging between 0.67 and 0.95) was indicative of the influence of other particle sources, with a finer size distribution, as suggested also by the daily trends at the stadium (Figure 3) and the reference station (Figure S5) at this location. Finally, the ratios for stadium #6 (0.12–0.20) were clear outliers and may not be interpreted comparably to those from the other 5 cities due to the shorter monitoring period (only 3 months for PM) at this African location.

The assessment of PM_2.5_/PM_10_ ratios and the identification of sources such as local dust re-suspension could facilitate the implementation of targeted mitigation strategies. For example, total or partial traffic bans could be implemented by city authorities in the surroundings of the stadia, in the days prior to and during competitions. This approach was already applied, at the city scale, during the Beijing Olympics in 2008 [21] (https://www.theguardian.com/world/2008/jul/21/china.olympicgames2008 (accessed on 6 February 2022)). In locations such as #2, with a larger impact of coarse particles during the dry season, another targeted strategy could be the application of dust binders for road dust [66,67,68], either around or inside the stadium itself, which could be implemented by stadium managers.

### 3.3. Similarity Analysis Using Self-Organising Maps (SOMs)

The data were further analysed using self-organising maps (SOMs), focusing on the inter-relationships between the air quality parameters monitored in each location. SOMs support source identification and the confirmation or challenging of the emission sources interpreted in the previous sections, as well as the characterisation of the dependencies between air pollutants and their interpretation in absence of reference data. Finally, when unexpected dependencies are detected, this may reveal the influence of monitoring artefacts or local phenomena. In this work, the SOMs analysis aimed to investigate commonalities in emission sources and expose additional air quality processes or mechanisms affecting each of the study areas. This analysis was not possible for stadium #6 due to the small size of the dataset.

**Figure 5:**
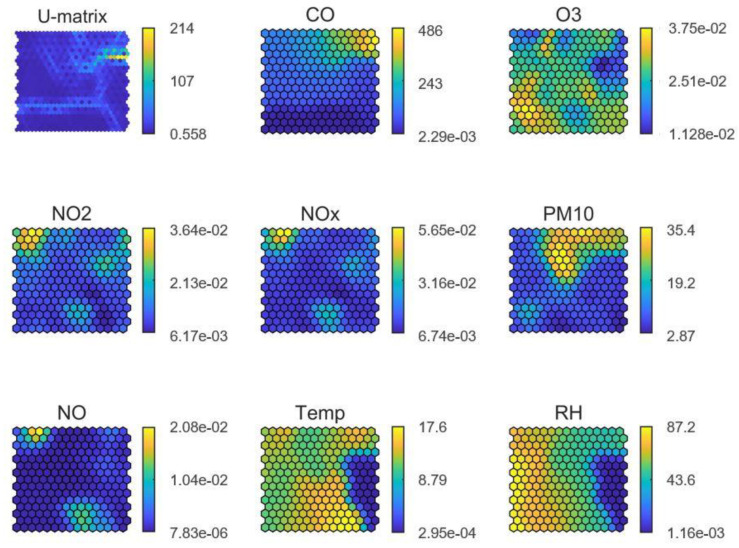
SOM of available data for stadium #1, in Europe. The colour scale is automatically adjusted among graphs and does not reflect the full range of values per parameter.

In stadium #1 (in Europe; Figure 5), high PM_10_ concentrations demonstrated strong association (i.e., they were classified in the same region of the SOM) with relatively low ambient temperature and moderate RH, partially overlapping with CO. This may suggest that PM_10_ shared a common emission source with CO, while other sources also influenced PM_10_ concentrations which seem to be unrelated to NO or NO_2_ emissions. Based on the daily pattern shown in Figure 5 for this stadium, the additional PM_10_ source(s) probably generated emissions in the afternoon hours (between 12–20 h). The O_3_ pattern, on the other hand, evidenced reaction with NO towards the production of NO_2_ (titration). The distribution of the high O_3_ values across the SOM, occurring when RH is high and temperature moderate to high, suggests the formation of O_3_ from precursors (NO_X_) and volatile organic compounds (VOCs), as well as the transport of this secondary pollutant from neighboring areas. As a result, the SOMs analysis for stadium #1 supported the validation of the data generated by the medium-cost monitor, as it revealed inter-relationships between pollutants (e.g., NO_2_ and O_3_) and atmospheric processes (e.g., titration) characteristic of urban environments.

**Figure 6:**
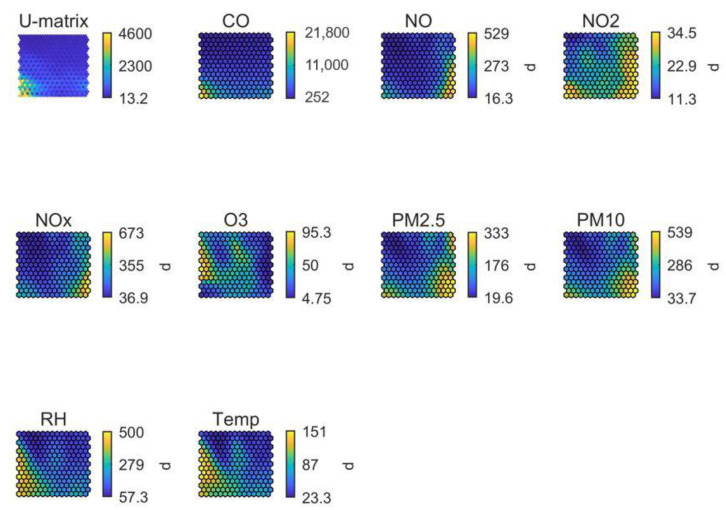
SOM of available data for stadium #2, in Africa. The colour scale is automatically adjusted among graphs and does not reflect the full range of values per parameter.

SOM analysis for stadium #2 (in Africa; Figure 6) showed that PM pollution levels were higher during the wetter season in comparison to the dryer season (as reflected by RH levels). High PM concentrations interrelated with high CO, NO and NO_2_ concentrations, suggesting mainly combustion sources as a key source. While the time series in Figure 3 evidence the influence from traffic, the impact of additional combustion sources requires further research. Temperature and RH increased in parallel (i.e., the same region of the SOM), demonstrating a characteristic African climatic pattern. On the other hand, O_3_ levels were driven by solar radiation as reflected by high temperature, with values not being influenced by NO, which was an unexpected result for an urban environment. This may indicate that (a) O_3_ formation was VOC-driven in this region, and/or that (b) regional transport of O_3_ could be a major source of O_3_ towards this urban area, with a lower influence of local-scale formation. Finally, while PM_10_ showed the highest concentrations in only one region of the SOM, PM_2.5_ concentrations were high in more than one region, indicating diverse air pollution sources or processes. This result was complementary to the conclusions from the daily air pollutant patterns (Figure 3). The SOM analysis for this stadium was especially valuable in terms of identifying the link between air pollutant concentrations and local meteorology.

**Figure 7:**
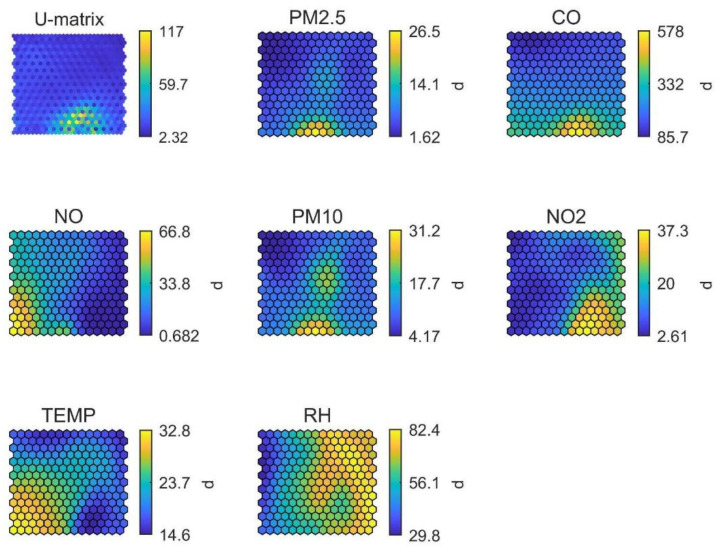
SOM of available data for stadium #3, in Oceania. The colour scale is automatically adjusted among graphs and does not reflect the full range of values per parameter.

In Oceania (stadium #3, Figure 7), a local emissions mechanism seems to be a key air pollutants source as evidenced by the correlation between high PM_10_, PM_2.5_ and CO (and slightly NO) concentrations, in the same region of the SOMs. Aside from this, the SOM revealed the possible overestimation of PM concentrations due to the interference of relative humidity, a known artefact affecting optical sensors [38]. This effect was identified by the high RH appearing together with increased PM levels in the central area of the SOM and should be considered as a limitation of this technology. However, RH was not the highest at this stadium when compared to other locations (Figure S4). Temperature did not peak in parallel with RH as in the case of stadium #2 in Africa, clearly demonstrating a different climatic background.

**Figure 8:**
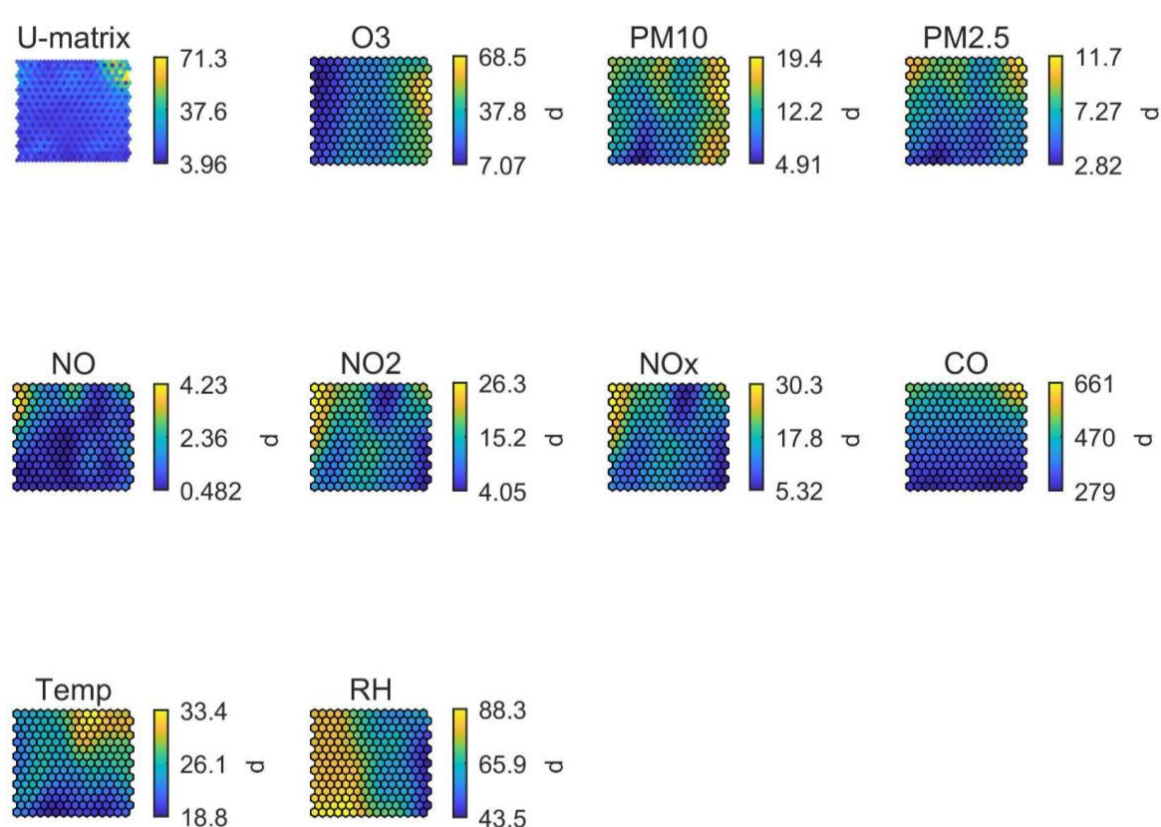
SOM of available data for stadium #5, in Asia. The colour scale is automatically adjusted among graphs and does not reflect the full range of values per parameter.

The SOM analysis for stadium #4 (in Asia; Figure 8) contributed to the interpretation of the sources of O_3_ in the region: maximum O_3_ concentrations did not coincide with high temperatures and thus did not seem to be driven by sunlight-triggered photochemistry, suggesting transport from other regions as the most probable origin. PM_2.5_ and PM_10_ levels did not seem to be influenced by high relative humidity at this location (contrarily to stadium #3), while they did evidence the influence from different emission sources.

**Figure 9:**
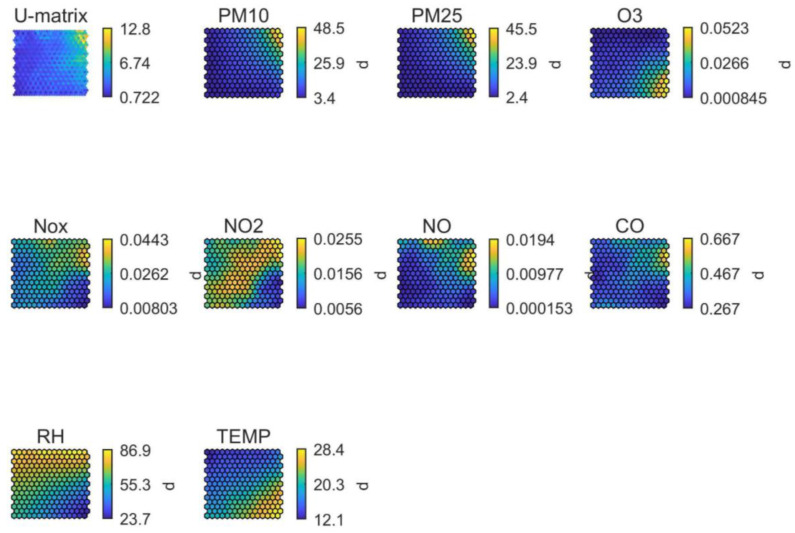
SOM of available data for stadium #6, in America. The colour scale is automatically adjusted among graphs and does not reflect the full range of values per parameter.

Finally, for stadium #5 (in America; Figure 9), SOMs indicated common emission sources for NO_2_ and PM but also the influence of additional processes for NO_2_. The fact that high CO and PM concentrations were not classified in the same area of the SOM confirmed not a single emission source was the only contributor to PM, as discussed above in Figure 3. This pattern could also be influenced by the poorer performance of the CO sensor. Sources controlling the daily patterns of these pollutants could be linked to the fine particle source described above during the analysis of the PM_2.5_/PM_10_ ratios (Figure 4). Interestingly, high O_3_ levels in the stadium did not seem to be associated with local conditions (absence of low O_3_ values in parallel with high NO_2_ and low NO values) but appeared instead during the lowest RH conditions, which were accompanied by the highest temperatures. This suggests photochemistry in the greater area as a potential mechanism leading to O_3_ production, which is valuable information in terms of the design and planning of the spatial scale of potential mitigation measures to reduce concentrations of this hazardous gaseous pollutant.

As a result, SOMs of the MAQM datasets allowed us to confirm the main interpretations obtained from the time series analysis (e.g., the influence of a local mechanism such as traffic as a key emission source in all stadia), as well as to deepen the analysis of the regional or local origin of certain of pollutants (e.g., O_3_ in stadia #4 vs. #5) and the links with meteorology (stadium #2), and to identify the influence of monitoring artefacts (stadium #3).

## 4. Conclusions

This work aimed to test the usability of sensor technologies in athletics stadia worldwide in order to test the usefulness of the information generated in different types of cities, e.g. where reference air quality data are available (city#1) vs. cities with no reference networks (city #2), or cities showing characteristic urban air quality patterns (city #1) vs. cities with more unexpected patterns (city #5). For this assessment the sensor systems were not calibrated locally, and instead relied on their initial calibration in which expected limitations such as the effect of environmental conditions or cross sensitivities are dealt with without relying on artificial intelligence models (independently of external or reference data). The limitations of sensor technologies (the ones tested in this work but also others) are well known and acknowledged in this work. For the specific application described in this study, we conclude that the data generated described daily air pollutant trends, revealing basic relationships among pollutants and identifying hourly peaks for the different pollutants under study. It was possible to identify local climatic patterns (in terms of temperature and relative humidity) which may impact local air quality and suggest common emission sources which contributed to air pollution levels in the vicinity of the areas of interest (via the time series and the SOM analysis). In addition, the PM_2.5_/PM_10_ ratios provided insights into potential emission sources impacting the exposure of athletes and visitors during sports competitions. The results from this work may be applicable for:

(a) Guidance for event organisers: hyper-local air quality monitoring in the stadia allows for the identification of periods of the day with the lowest average relative pollutant concentrations. Further research is necessary to identify the exact value range which reference instruments would have reported, as well as the specific air pollutants that may trigger or exacerbate respiratory conditions typical of the athlete community (e.g., asthma or exercise-induced-bronchospasm; [13]).
(b) Guidance for competitions: setting up guidelines and/or air pollutant thresholds would help minimise air pollution exposures for athletes and avoid inequalities in training/competing conditions in different parts of the world, by deciding on the potential cancellation or postponement of events. A similar work was proposed for urban marathons [26].
(c) Guidance for mitigation: certain mitigation actions could be implemented inside the stadia (e.g., application of dust binders). Measures targeting traffic could be implemented by city authorities (e.g., total or partial bans during events), while those targeting regional-scale O_3_, as identified using the SOMs, would require coordination of city and regional stakeholders.

Finally, the limitations of this work should be considered. The main limitation is the lack of local calibration of the air quality monitors, which is at the same time an intrinsic characteristic of this study design. As a result, the extent to which the local aerosol mix and meteorology impacted the sensor readings is unknown, although it is expected that there was at least influence from ambient relative humidity, particle density and particle hygroscopicity.

## Figures and Tables

**Table 1:** Location, monitoring dates and number of valid datapoints (Nr. Data) for each of the air quality monitors deployed. Local network: indicates whether a local air quality network was operational in the city.

Location	Stadium#1	Stadium#2	Stadium#3	Stadium#4	Stadium#5	Stadium#6
Continent	Europe	Africa	Oceania	Asia	America	Africa
Hemisphere	North	North	South	North	North	South
Start	Dec. 2018	Dec. 2018	Jan. 2019	May. 2019	Feb. 2019	Aug. 2020
End	Nov. 2019	Dec. 2019	Oct. 2019	Dec. 2019	Nov. 2019	Dec. 2020
Nr. Data: NO	5909	3643	3580	5044	7270	3606
Nr. Data: NO_2_	5907	3628	3582	5044	7270	3606
Nr. Data: O_3_	5900	3628	2707	5044	7270	3606
Nr. Data: PMx	5910	3643	3582	3472	7001	1818
Nr. Data: CO	5910	3644	3582	5044	7270	3606
Nr. Data: T	5910	3643	3582	5044	7270	3606
Nr. Data: RH	5910	3643	3582	5044	7270	3606
Local network	Yes	No	Yes	Yes	Yes	No

## Data Availability

Not applicable.

## Supplementary Materials

The following supporting information can be downloaded at: https://www.mdpi.com/article/10.3390/ijerph19063561/s1, Figure S1: Intercomparison between monitor #1 and EU-reference data for gaseous pollutants at the Barcelona (Palau Reial) reference station; Figure S2: Intercomparison between monitor #5 and reference data for PM_10_ and PM_2.5_ at a reference station in city #5; Figure S3: Mean daily evolution of ambient temperature for the months July–September 2019, for the 6 stadia in major cities in: #1: Europe, #2: Africa, #3: Oceania; #4: Asia; #5: America; #6: Africa (data from October–December 2020); Figure S4: Mean daily evolution of ambient relative humidity for the months July–September 2019, for the 6 stadia in major cities in: #1: Europe, #2: Africa, #3: Oceania; #4: Asia; #5: America; #6: Africa (data from October–December 2020); Figure S5: Mean daily evolution of PM_10_ (red) and PM_2.5_ (blue) for the months July–September 2019, for an official reference station in city #5 located at approximately 20 km from the stadium where the monitor was located.
