# Supplementary material for: Air Quality Sensors Systems as Tools to Support Guidance in Athletics Stadia for Elite and Recreational Athletes
Source: Int J Environ Res Public Health. 2022 Mar 17;19(6):3561. doi: 10.3390/ijerph19063561 (PMC8950704; doi:10.3390/ijerph19063561)
Supplement: Supplementary file 1 [file ijerph-19-03561-s001.zip › ijerph-1608553-SI.pdf]

International Journal of
Environmental Research and Public Health

MDPI

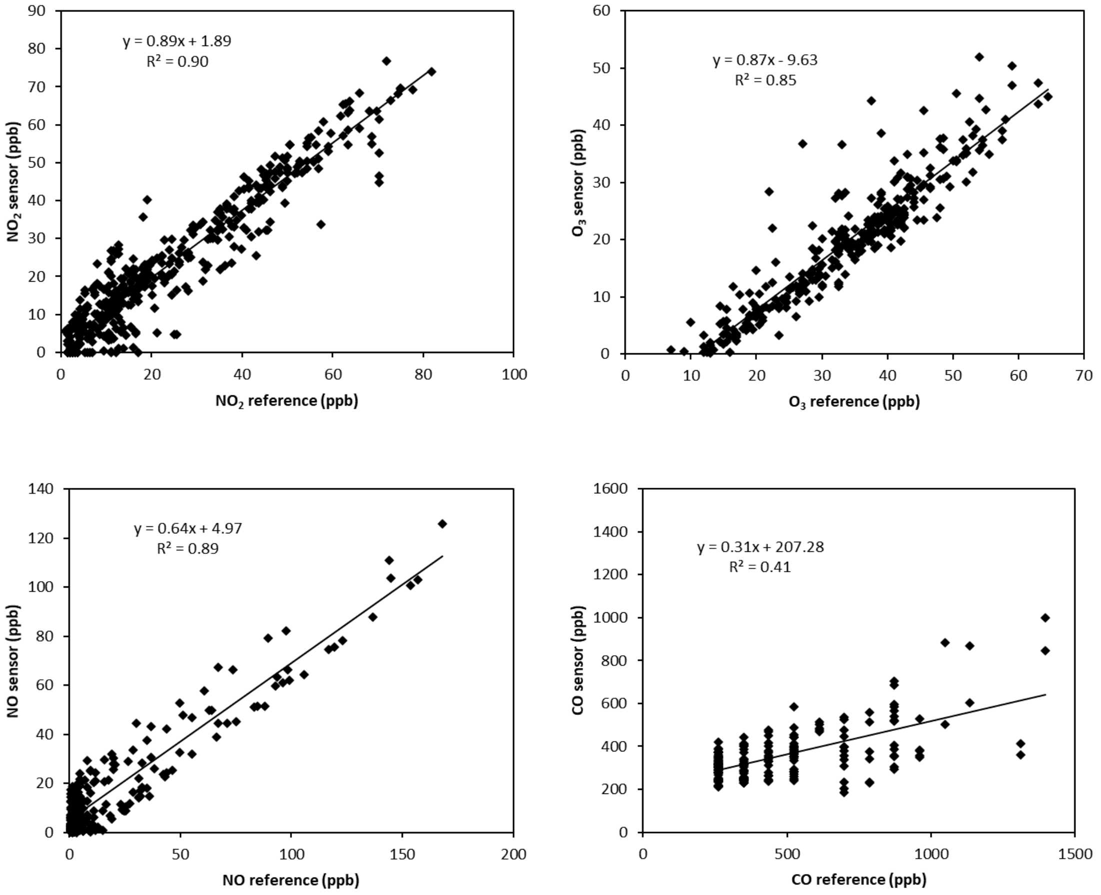

Figure S1. Intercomparison between monitor #1 and EU-reference data for gaseous pollutants at the Barcelona (Palau Reial) reference station.

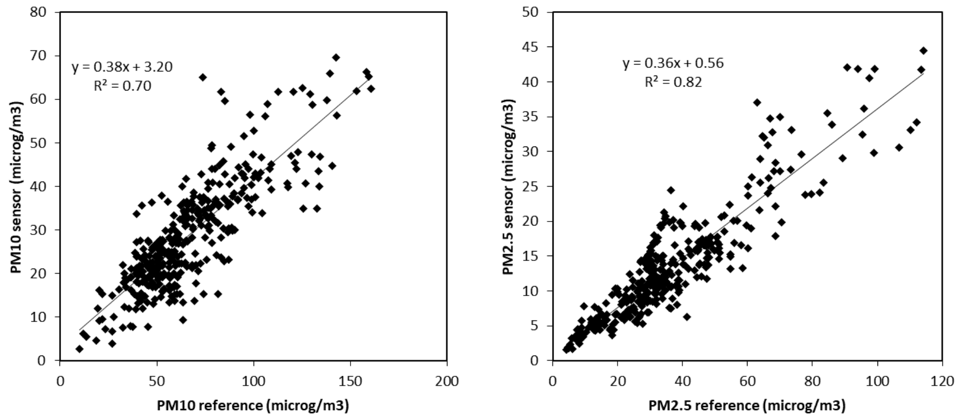

Figure S2. Intercomparison between monitor #5 and reference data for PM_{10} and $PM_{2.5}$ at a reference station in city #5.

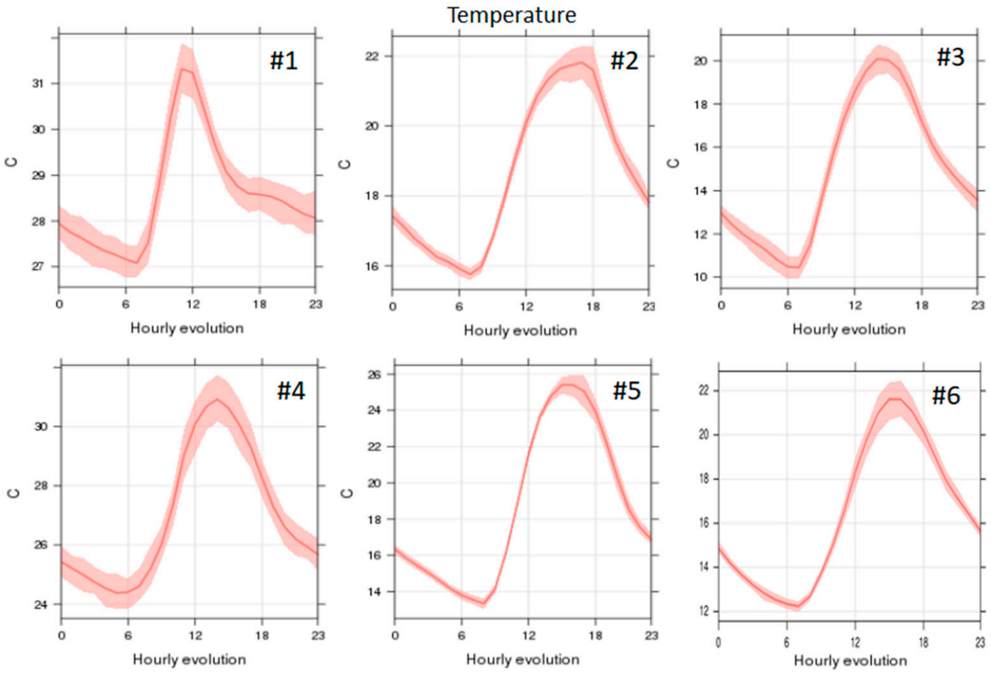

Figure S3. Mean daily evolution of ambient temperature for the months July-September 2019, for the 6 stadia in major cities in: #1: Europe, #2: Africa, #3: Oceania; #4: Asia; #5: America; #6: Africa (data from October-December 2020).

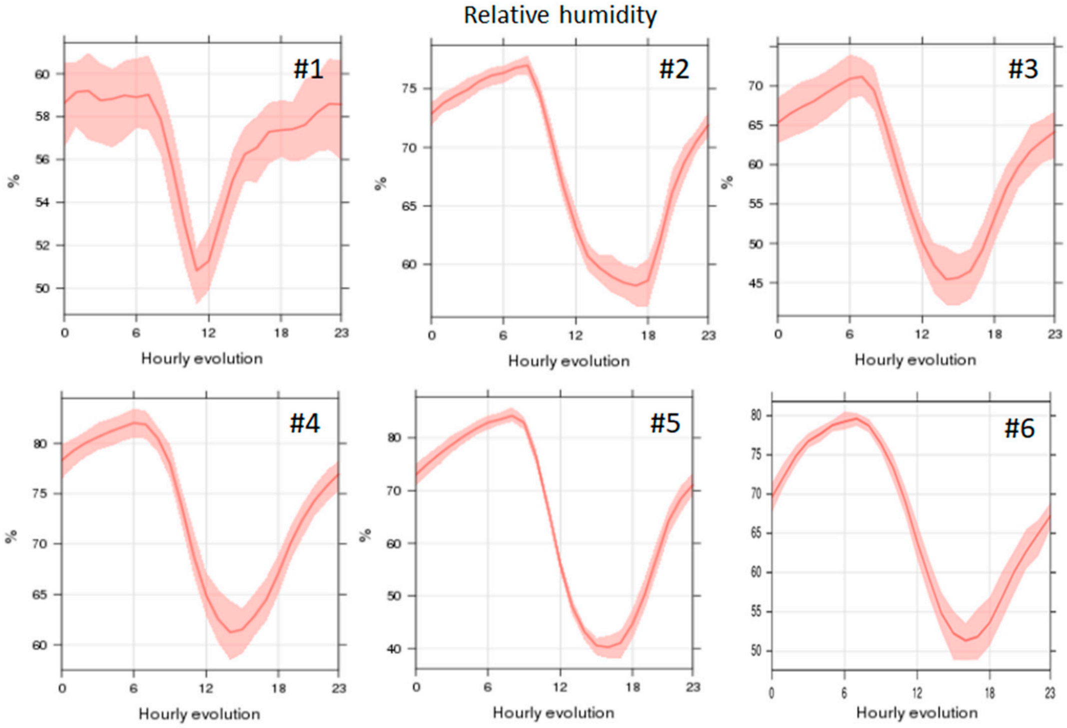

Figure S4. Mean daily evolution of ambient relative humidity for the months July-September 2019, for the 6 stadia in major cities in: #1: Europe, #2: Africa, #3: Oceania; #4: Asia; #5: America; #6: Africa (data from October-December 20200).

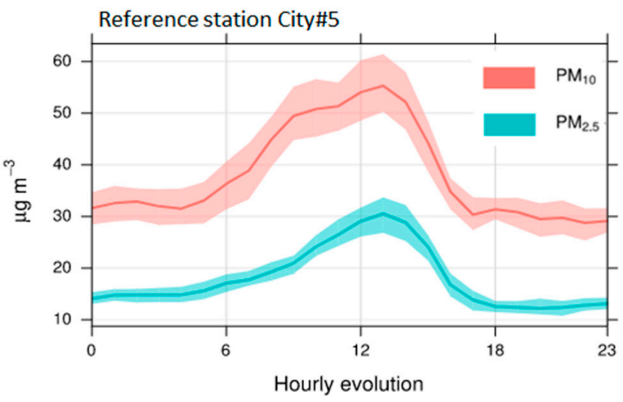

Figure S5. Mean daily evolution of PM_{10} (red) and $PM_{2.5}$ (blue) for the months July-September 2019, for an official reference station in city #5 located at approximately 20 km from the stadium where
